# Association between body mass index at diagnosis and outcomes in Chinese children with newly diagnosed acute lymphoblastic leukemia

**DOI:** 10.1002/cam4.5188

**Published:** 2022-09-27

**Authors:** Wenting Hu, Yin Ting Cheung, Yanjing Tang, Li Hong, Yuan Zhu, Jing Chen, Zhuo Wang, Min Zhou, Yijin Gao, Jing Chen, Benshang Li, Huiliang Xue, Longjun Gu, Shuhong Shen, Jingyan Tang, Ching‐Hon Pui, Hiroto Inaba, Jiaoyang Cai

**Affiliations:** ^1^ Department of Hematology/Oncology, Key Laboratory of Pediatric Hematology and Oncology of China Ministry of Health, National Children's Medical Center, Shanghai Children's Medical Center Shanghai Jiao Tong University School of Medicine Shanghai China; ^2^ School of Pharmacy, Faculty of Medicine The Chinese University of Hong Kong Hong Kong China; ^3^ Department of Clinical Nutrition, Shanghai Children's Medical Center Shanghai Jiao Tong University School of Medicine Shanghai China; ^4^ Department of Oncology St. Jude Children's Research Hospital Memphis Tennessee USA; ^5^ Department of Global Pediatric Medicine St. Jude Children's Research Hospital Memphis Tennessee USA

**Keywords:** acute lymphoblastic leukemia, body mass index, pediatrics, treatment‐related mortality

## Abstract

**Purpose:**

Studies of the association between body mass index (BMI) at diagnosis and treatment outcome in children with acute lymphoblastic leukemia (ALL) have yielded inconsistent results. Hence, we conducted a retrospective study in a large cohort of Chinese children with ALL treated with contemporary protocols.

**Patients and Methods:**

A total of 1437 children (62.1% male; median age at diagnosis 5.7 years, range: 2.3–16.3 years) were enrolled in two consecutive clinical trials at the Shanghai Children's Medical Center. The rates of overall survival, event‐free survival, relapse, treatment‐related mortality, and adverse events were compared among patients who were underweight (BMI < 5th percentile), at a healthy weight (5th to 85th percentile), overweight (>85th to <95th percentile), and obese (≥95th percentile).

**Results:**

At diagnosis, 91 (6.3%) patients were underweight, 1070 (74.5%) were at a healthy weight, 91 (6.3%) were overweight, and 185 (12.9%) were obese. No significant association was found between weight status and 5‐year overall survival, event‐free survival, or relapse in the overall cohort. When analyzed as a continuous variable, a higher BMI Z‐score was associated with treatment‐related mortality (hazard ratio 1.33 (95% confidence interval [CI], 1.05–1.68%), *p* = 0.02). The treatment‐related mortality rate was higher in the overweight (5.5%, 95% CI 0.8–10.2%) and obese (3.2%, 95% CI 0.6–5.8%) groups compared with the underweight (0.0%) and healthy‐weight groups (1.9%, 95% CI 1.1–2.7%; *p* = 0.04). Multivariable analysis showed that children who were overweight had a higher risk of treatment‐related mortality (hazard ratio 3.8, 95% CI 1.3–11.4).

**Conclusion:**

While body weight status was not associated with event‐free survival or overall survival, overweight patients were at higher risk of treatment‐related mortality.

## Abstract 2

We found that overweight/obese children were at higher risk of treatment‐related mortality and developing severe toxicities than underweight or health‐weight children. Our findings reinforce the importance of the normalizing weight during the treatment period to improve survival and treatment outcomes.

## 1 INTRODUCTION

The impact of body mass index (BMI) on the outcome of children with acute lymphoblastic leukemia (ALL) has been evaluated in several studies. In the initial study of Children's Oncology Group (COG) of patients enrolled between 1988 and 1995, the 343 obese patient had significantly worse 5‐year event‐free survival (EFS) (72% vs. 77%) than the 3071 nonobese patients.^1^ The adverse effect of obesity was especially prominent among patients ≥10 years old at diagnosis who were significantly more likely to be Hispanic.^1^ In a subsequent COG study of children with high‐risk ALL enrolled between 1996 and 2002, the 5‐year EFS rates of the 279 obese (64%) and the 117 underweight patients (65%) were significantly worse than that of the 1612 normal or overweight patients (74%).^2^ However, two studies from St. Jude Children's Research Hospital (SJCRH) between 1988 and 2000 and between 2000 and 2007 showed that the 5‐year EFS did not differ significantly when patients were categorized to underweight, healthy weight, overweight, and obese.^3^, ^4^ Among patients enrolled in the Dutch Childhood Oncology Group‐ALL9 protocol between 1997 and 2004, the underweight patients had 2‐fold higher risk of relapse but no significant difference in EFS as compared to the other 679 patients who were not underweight.^5^ These seemingly discrepant results among the studies may be due to difference in treatment, proportion of patients with various racial groups or ancestries, or small number of underweight or overweight/obese patients studied. Few studies have evaluated the impact of BMI at diagnosis on leukemia outcomes in Asian children, and none have addressed the Chinese population. To address these gaps, we conducted a retrospective study to evaluate the association between BMI at diagnosis and treatment outcomes in a relatively large cohort of Chinese children with ALL who were enrolled in two consecutive protocols.

## 2 METHODS

### 2.1 Patients

This retrospective study was conducted in a cohort of patients with pediatric ALL who were treated at the Shanghai Children's Medical Center (SCMC) in China. This study was approved by the Research Ethics Board at SCMC. Written informed consent was obtained from the parents or legal guardians of all patients.

Children aged 2 to 18 years who were newly diagnosed with ALL between May 1, 2005 and June 30, 2018 were included in the study. Patients who were diagnosed before December 31, 2014 were treated on the SCMC‐ALL‐2005 protocol.^6^, ^7^ Patients who were diagnosed from January 1, 2015 to June 30, 2018 were treated on the Chinese Children's Cancer Group ALL‐2015 (CCCG‐ALL‐2015) protocol (clinical trial registration number: ChiCTR‐IPR‐14005706).^8^, ^9^ The CCCG‐ALL‐2015 protocol is a prospective, risk‐stratified, and minimal residual disease (MRD)‐directed treatment modified from the SCMC‐ALL‐2005 study and the St. Jude Children's Research Hospital Total Therapy 15 and 16 studies.^10^, ^11^ Risk classification in CCCG‐ALL‐2015 protocol was based on the presenting characteristics (age, white blood cell count, immunophenotype, and cytogenetics) and treatment response as measured by MRD levels during remission induction and at the end of remission induction therapy. Native L‐asparaginase was used in the SCMC‐ALL‐2005 protocol but PEG‐Asparaginase (PEG‐ASP) was used to replace L‐asparaginase in the CCCG‐ALL‐2015 protocol.

Patients younger than 2 years of age at diagnosis were excluded from this study due to the limited diagnostic accuracy of BMI percentile calculation for this age group. Patients lacking data for BMI at diagnosis were also excluded.

### 2.2 Data extraction

All study data were retrieved from in‐house electronic databases. The data of patients treated with the SCMC‐ALL‐2005 protocol were retrieved from the Pediatric Oncology Network Database (POND; http://www.pond4kids.org), an online pediatric oncology database developed by St. Jude Children's Research Hospital. The data of patients treated with the CCCG‐ALL‐2015 protocol were retrieved from the Leukemia Registry and Management System of the National Children's Medical Center. Both databases include patient‐specific data, such as clinical diagnosis, adverse events, follow‐up, morbidities and mortality, and information on hospitalization. The databases have been proven to be reliable sources of health administrative data for conducting epidemiological research in China.^6^, ^9^

### 2.3 BMI calculation and classification

BMI was calculated by dividing the body weight in kilograms by the square of height in meters (kg/m^2^). It is well established that the thresholds for overweight and obesity in children are defined differently in Western and Asian populations.^12^ Therefore, BMI percentiles were determined using BMI growth curves for Chinese children and adolescents aged 0–18 years instead of growth charts from the World Health Organization (WHO) or the United States Centers for Disease Control and Prevention (Figure S1).^13^, ^14^ Based on the BMI at diagnosis, patients were classified according to age‐adjusted BMI percentiles. Healthy weight was defined as a BMI from the 5th to the 85th percentile, underweight under the 5th percentile, overweight between the 85th and 95th percentiles, and obese greater than or equal to the 95th percentile.^15^ In addition, BMI was evaluated as a continuous variable by converting the BMI percentiles into Z‐scores using WHO Anthro and Anthroplus calculator to examine its relationship with outcomes.

### 2.4 Study outcomes

EFS was calculated from the date of diagnosis to the first major adverse event, including induction failure, relapse, death, second malignant neoplasm, and physician‐directed deviation from the treatment protocol. A decision to abandon treatment by the parents or transfer to another hospital was considered censoring events. Overall survival (OS) was calculated from the date of diagnosis to death from any cause or the last follow‐up.

### 2.5 Statistical analysis

Descriptive statistics (frequencies, medians, and interquartile ranges) were used to summarize the patient characteristics of the cohort. EFS and OS curves were estimated according to the Kaplan–Meier method, and the log‐rank test was used to compare survival among different BMI groups (underweight vs. healthy‐weight vs. overweight vs. obese). Confidence intervals (CIs) for effects of different BMI groups were calculated.

The chi‐square test was used to detect differences between BMI categories, stratified by well‐documented risk factors for poorer outcomes in ALL. These factors included older age, higher white blood cell count, CNS involvement, T‐cell immunophenotype, presence of specific cytogenetics abnormalities, MRD by the end of induction and protocol.^6^, ^10^, ^11^ The cumulative incidence functions of relapse, treatment‐related mortality, or risk of grade 4–5 infections were estimated by the Kalbfleisch–Prentice method and compared using Gray's test.^16^, ^17^ Any relapse, death, second malignancy, or off‐protocol treatment decided by the treating physician was considered a competing event. Multivariate regression analyses were performed to determine independent factors associated with EFS, OS, and treatment‐related mortality using Fine–Gray regression models to estimate the hazard ratio (HR). Fisher's exact test was used to analyze grade 4–5 toxicities in different BMI groups.

All statistical analyses were performed using R statistical software version 3.4.4. For all tests, a *p* value <0.05 (two‐sided test) was considered statistically significant.

## 3 RESULTS

### 3.1 Study population

Of the 1492 patients aged 2 to 18 years who were enrolled in the SCMC‐ALL‐2005 or CCCG‐ALL‐2015 protocol, BMI data were available for 1437 (96.3%) patients. Among these 1437 patients, the median age at diagnosis was 5.1 years (range: 2.0–17.9 years), and 62.1% (893/1437) were male. Outcome data were updated on November 30, 2021; the median follow‐up time was 6.3 years (interquartile range 4.0–9.5, range: 0.1–15.7 years). Two‐thirds (64.0%) of the patients were treated on the SCMC‐ALL‐2005 protocol, while the others (36.0%) were treated on the CCCG‐ALL‐2015 protocol. The majority of patients (97.2%) achieved complete remission after induction therapy. At diagnosis, 91 (6.3%) patients were underweight, 1070 (74.5%) were at a healthy weight, 91 (6.3%) were overweight, and 185 (12.9%) were obese. The median Z‐score was 0.2 (range − 5.7 to 5.6). Table 1 provides the patient characteristics according to BMI group. We found no differences in clinical characteristics among BMI groups, with the exception that more obese patients were enrolled in the SCMC‐ALL‐2005 protocol than in the CCCG‐ALL‐2015 protocol (14.3% vs. 10.4%, *p* = 0.03).

**TABLE 1:** Patient characteristics according to body mass index group

Variable	Total *N* (%)	Underweight *N* (%)	Healthy weight *N* (%)	Overweight *N* (%)	Obese *N* (%)	*p* Value
*n*	1437	91 (6.3%)	1070 (74.5%)	91 (6.3%)	185 (12.9%)	
Final Risk group						0.50
Low	637 (44.3)	46 (7.2)	473 (74.3)	42 (6.6)	76 (11.9)	
Intermediate/High	800 (55.7)	45 (5.6)	597 (74.6)	49 (6.1)	109 (13.6)	
Age at diagnosis (median, range)		5.7 (2.3–16.3)	5.0 (2.0–17.9)	5.2 (2.0–16.0)		0.30
<10 year	1185 (82.5)	69 (5.8)	887 (74.9)	73 (6.2)	156 (13.2)	
≥10 years	252 (17.5)	22 (8.7)	183 (72.6)	18 (7.1)	29 (11.5)	
Sex						0.07
Male	893 (62.1)	60 (6.7)	645 (72.2)	65 (7.3)	123 (13.8)	
Female	544 (37.9)	31 (5.7)	425 (78.1)	26 (4.8)	62 (11.4)	
Leukocyte count (×10^9^/L)						0.21
<50	1206 (83.9)	83 (6.9)	895 (74.2)	77 (6.4)	151 (12.5)	
≥50	231 (16.1)	8 (3.5)	175 (75.8)	14 (6.1)	34 (14.7)	
CNS status						0.34
CNS1	1300 (90.5)	84 (6.5)	974 (74.9)	80 (6.2)	162 (12.5)	
Other CNS status	137 (9.5)	7 (5.1)	96 (70.1)	11 (8.0)	23 (16.8)	
Immunophenotype						0.21
B‐lineage	1257 (88.4)	81 (6.4)	944 (75.1)	79 (6.3)	153 (12.2)	
T‐lineage	180 (11.6)	10 (5.6)	126 (70.0)	12 (6.7)	32 (17.8)	
t (9;22)(*BCR‐ABL1*)						0.93
Present	50 (3.5)	2 (4.0)	40 (80.0)	2 (4.0)	6 (12.0)	
Absent	1387 (96.5)	89 (6.4)	1030 (74.3)	89 (6.4)	179 (12.9)	
t (1;19)(*TCF3‐PBX1*)						0.43
Present	52 (3.6)	4 (7.7)	42 (80.8)	3 (5.8)	3 (5.8)	
Absent	1385 (96.4)	87 (6.3)	1028 (74.2)	88 (6.4)	182 (13.1)	
t (12;21)(*ETV6‐RUNX1*)						0.95
Present	263 (18.3)	18 (6.8)	193 (73.4)	17 (6.5)	35 (13.3)	
Absent	1174 (81.7)	73 (6.2)	877 (74.7)	74 (6.3)	150 (12.8)	
Complete remission						0.12
Yes	1397 (97.2)	90 (6.4)	1042 (74.6)	90 (6.4)	175 (12.5)	
No	40 (2.8)	1 (2.5)	28 (70.0)	1 (2.5)	10 (25.0)	
MRD by the end of induction^a^						0.06
<0.01%	1121 (85.4)	74 (6.6)	826 (73.7)	83 (7.4)	138 (12.3)	
≥0.01%	191 (14.6)	13 (6.8)	152 (79.6)	5 (2.6)	21 (11.0)	
Study protocol						0.03
SCMC‐ALL‐2005	919 (64.0)	56 (6.1)	684 (74.4)	48 (5.2)	131 (14.3)	
CCCG‐ALL‐2015	518 (36.0)	35 (6.8)	386 (74.5)	43 (8.3)	54 (10.4)	

Abbreviations: CNS, central nervous system; MRD, minimal residual disease.

^a^ MRD test timing is on day 55 for patients treated with SCMC‐ALL‐2005 protocol, and on day 46 for patients treated with CCCG‐ALL‐2015 protocol.

### 3.2 BMI and ALL outcomes

**FIGURE 1:**
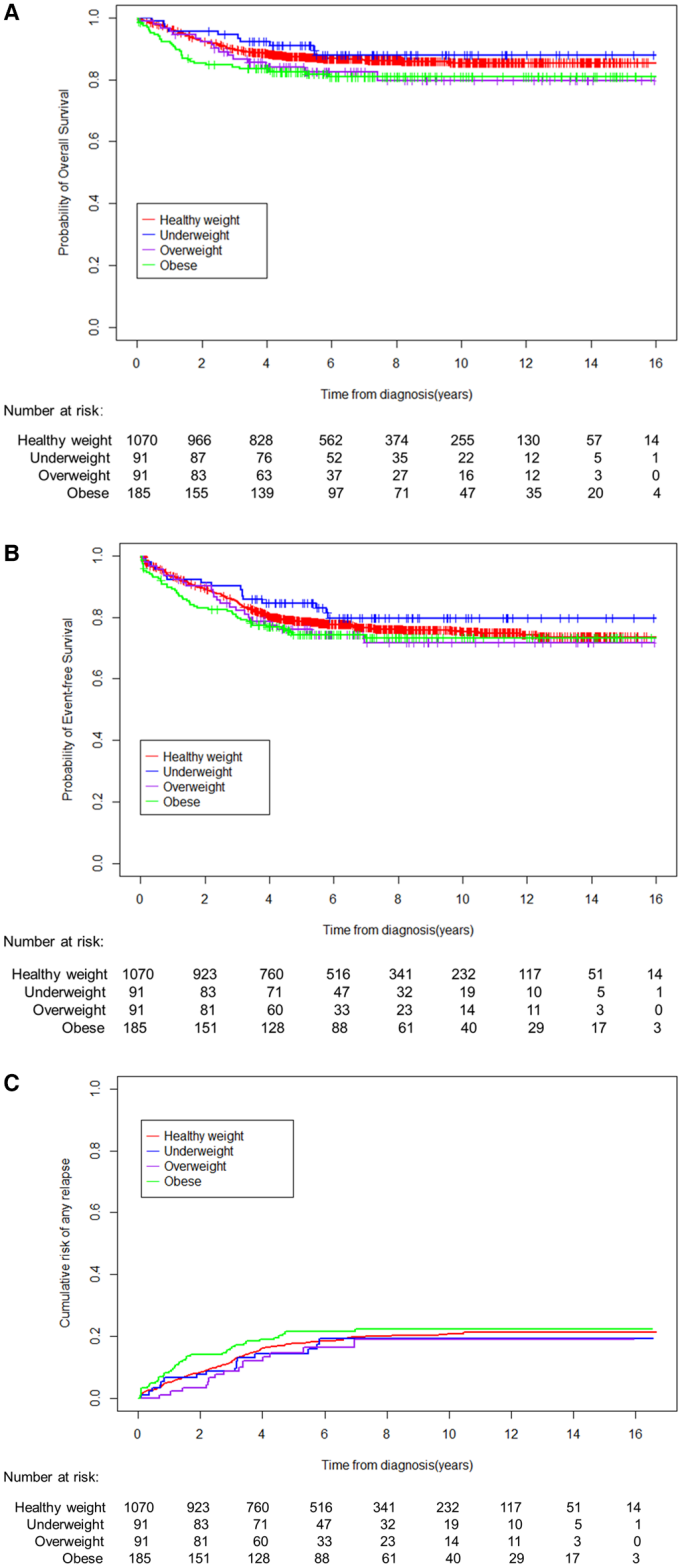
Overall survival (A), event‐free survival (B), and cumulative risk of any relapse (C) in 1437 children with acute lymphoblastic leukemia across different body mass index (BMI) groups.

The 5‐year OS was lower in overweight and obese patients (84.1% [95% CI, 76.8–92.1%] and 82.4% [95% CI, 77.0–88.1%], respectively) than in the other groups, although the estimates were not significantly different from healthy‐weight (87.3% [95% CI, 85.3–89.4%]) or underweight patients (91.0% [95% CI, 85.3–97.2%]; *p* = 0.10; Figure 1A). The 5‐year EFS in healthy weight, underweight, overweight, and obese patients were 78.5% [95% CI, 76.0–81.1%], 84.6% [95% CI, 77.4–92.3%], 76.1% [95% CI, 67.6–85.6%], and 74.3% [95% CI, 68.1–81.0%], respectively (*p* = 0.50, Figure 1B). There were no significant differences in the 5‐year cumulative risk of any relapse (17.7% [95% CI, 15.4–20.0%] vs. 14.3% [95% CI, 7.0–21.6%] vs. 14.8% [95% CI, 7.3–22.3%] vs. 21.5% [95% CI, 15.5–27.5%]; *p* = 0.70; Figure 1C) between healthy‐weight, underweight, overweight, and obese patients. There were also no significant differences in EFS and overall among various BMI groups when the analyses were performed separately for patients treated in the SCMC‐ALL‐2005 and CCCG‐ALL‐2015 protocols, respectively (Table 2).

**TABLE 2:** Treatment outcomes according to selected clinical and biological characteristics

Variable	Total *N* (%)	5‐year event‐free survival % (95% CI)					5‐year overall survival % (95% CI)				
		Underweight	Healthy weight	Overweight	Obese	*p* Value	Underweight	Healthy weight	Overweight	Obese	*p* Value
Final risk group											
Low	637 (44.3)	89.1 (80.6–98.6)	88.0 (85.0–91.0)	85.5 (75.4–96.9)	84.5 (76.7–93.4)	0.80	97.6 (93.0–100)	94.2 (92.1–96.4)	88.0 (78.7–98.4)	93.2 (87.6–99.1)	0.20
Intermediate/High	800 (55.7)	79.9 (69.0–92.6)	70.9 (67.3–74.8)	67.9 (55.6–82.9)	67.1 (58.7–76.7)	0.50	84.4 (74.5–95.7)	81.7 (78.6–84.9)	80.8 (70.2–93.0)	74.8 (67.0–83.5)	0.30
Age at diagnosis (median, range)											
<10 year	1185 (82.5)	85.4 (77.4–94.2)	81.1 (78.5–83.8)	79.9 (71.0–89.9)	75.3 (68.7–82.6)	0.60	93.9 (88.4–99.9)	89.3 (87.3–91.4)	86.1 (78.4–94.5)	84.3 (78.7–90.3)	0.10
≥10 years	252 (17.5)	81.8 (67.2–99.6)	66.0 (59.4–73.4)	61.1 (42.3–88.3)	69.0 (54.0–88.0)	0.50	81.8 (67.2–99.6)	77.3 (71.3–83.7)	75.0 (56.0–100)	72.4 (57.8–90.7)	0.90
Sex											
Male	893 (62.1)	81.7 (72.4–92.1)	74.9 (71.5–78.4)	74.1 (63.8–86.0)	72.6 (64.8–81.2)	0.90	89.6 (82.1–97.9)	85.7 (83.0–88.5)	82.2 (73.1–92.4)	82.5 (76.0–89.6)	0.40
Female	544 (37.9)	90.2 (80.3–100)	84.0 (80.5–87.7)	80.8 (67.0–97.4)	77.4 (67.6–88.5)	0.40	93.5 (85.3–100)	89.7 (86.8–92.7)	88.5 (77.0–100)	82.0 (72.9–92.3)	0.40
Leukocyte count (×10^9^/L)											
<50	1206 (83.9)	86.7 (79.6–94.3)	81.3 (78.7–84.0)	74.6 (65.3–85.2)	78.0 (71.5–85.1)	0.30	93.8 (88.6–99.2)	89.0 (86.9–91.1)	83.0 (75.0–91.9)	86.5 (81.1–92.2)	0.10
≥50	231 (16.1)	62.5 (36.5–100)	64.0 (57.1–71.8)	85.1 (68.0–100)	58.1 (43.5–77.7)	0.40	62.5 (36.5–100)	78.4 (72.4–84.9)	90.0 (73.2–100)	64.1 (49.6–82.7)	0.06
CNS status											
CNS1	1300 (90.5)	85.7 (78.5–93.5)	78.6 (76.0–81.3)	75.7 (66.8–85.9)	73.8 (67.2–81.1)	0.30	91.5 (85.7–97.7)	87.4 (85.3–89.6)	82.1 (74.0–91.1)	81.7 (75.9–88.0)	0.05
Other CNS status	137 (9.5)	71.4 (44.7–100)	77.3 (69.2–86.4)	79.5 (57.7–100)	77.4 (61.8–97.1)	0.90	85.7 (63.3–100)	86.4 (79.8–93.5)	100	87.0 (74.2–100)	0.70
Immunophenotype											
B‐lineage	1257 (88.4)	86.3 (79.2–94.2)	80.0 (77.5–82.7)	76.4 (67.4–86.6)	75.0 (68.2–82.3)	0.40	92.4 (86.7–98.4)	89.0 (87.0–91.1)	83.2 (75.3–92.0)	82.7 (76.8–89.0)	**0.03**
T‐lineage	180 (11.6)	70.0 (46.7–100)	66.9 (59.0–75.8)	74.1 (52.6–100)	71.5 (57.3–89.1)	0.99	80.0 (58.7–100)	74.6 (67.2–82.8)	90.9 (75.4–100)	81.2 (68.8–96.0)	0.60
t (9;22) (*BCR‐ABL1*)											
Present	50 (3.5)	50.0 (12.5–100)	44.8 (31.4–63.9)	50.0 (12.5–100)	0	<**0.001**	50.0 (12.5–100)	66.1 (62.7–83.0)	50.0 (12.5–100)	0	**<0.001**
Absent	1387 (96.5)	85.3 (78.3–93.0)	79.8 (77.3–82.4)	76.7 (68.2–86.2)	76.8 (70.7–83.4)	0.60	91.9 (86.4–97.9)	88.1 (86.1–90.2)	85.1 (78.0–92.9)	85.1 (80.0–90.6)	0.30
t (1;19) (*TCF3‐PBX1*)											
Present	52 (3.6)	100	76.2 (64.3–90.2)	100	100	0.40	100	80.6 (69.4–93.6)	100	100	0.50
Absent	1385 (96.4)	83.8 (76.4–92.0)	78.6 (76.1–81.2)	75.2 (66.6–85.1)	73.8 (67.6–80.7)	0.50	90.6 (84.6–97.0)	87.6 (85.5–89.7)	83.6 (76.0–91.8)	82.1 (76.6–87.9)	0.08
t (12;21) (*ETV6‐RUNX1*)											
Present	263 (18.3)	88.9 (75.5–100)	87.6 (82.9–92.6)	81.6 (64.7–100)	79.6 (67.2–94.3)	0.40	94.1 (83.6–100)	95.2 (92.1–98.3)	94.1 (83.6–100)	91.4 (82.6–100)	0.80
Absent	1174 (81.7)	83.5 (75.4–92.5)	76.5 (73.7–79.4)	74.8 (65.4–85.7)	73.2 (66.3–80.8)	0.40	90.3 (83.7–97.4)	85.5 (83.2–88.0)	81.8 (73.2–91.3)	80.2 (74.0–86.9)	0.10
MRD by the end of induction^a^											
<0.01%	1121 (85.4)	86.4 (78.9–94.6)	83.9 (81.4–86.5)	79.9 (71.6–89.3)	81.2 (74.8–88.1)	0.70	93.1 (87.4–99.1)	91.5 (89.6–93.5)	87.5 (80.5–95.1)	89.0 (83.8–94.4)	0.40
≥0.01%	191 (14.6)	76.9 (57.1–100)	60.1 (52.5–68.8)	30.0 (6.3–100)	61.9 (44.3–86.6)	0.70	84.6 (67.1–100)	78.1 (71.6–85.3)	60.0 (29.3–100)	70.7 (53.5–93.6)	0.20
Study protocol											
SCMC‐ALL‐2005	919 (64.0)	80.4 (70.6–91.5)	75.3 (72.1–78.7)	72.6 (61.0–86.5)	69.6 (62.0–78.0)	0.70	87.5 (79.3–96.6)	83.5 (80.8–86.4)	81.1 (70.7–93.0)	77.6 (70.7–85.1)	0.30
CCCG‐ALL‐2015	518 (36.0)	91.4 (82.6–100)	84.4 (80.7–88.2)	81.0 (70.0–93.8)	86.7 (77.9–96.4)	0.60	96.4 (89.8–100)	94.2 (91.8–96.6)	88.1 (78.8–98.5)	94.4 (88.5–100)	0.30

Abbreviations: CNS, central nervous system; MRD, minimal residual disease.

a MRD test timing is on day 55 for patients treated with SCMC‐ALL‐2005 protocol, and on day 46 for patients treated with CCCG‐ALL‐2015 protocol.

When analyzed as a continuous variable, a higher BMI Z‐score was associated with poorer OS (HR: 1.14 [95% CI, 1.02–1.27%], *P* = 0.02) and marginally poorer EFS (HR: 1.08 [95% CI, 0.99–1.17%], *p* = 0.07). An increase in BMI Z‐score was also associated with a higher hazard of treatment‐related mortality (HR: 1.33 [95% CI, 1.05–1.68%], *p* = 0.02). We did not observe significant association between BMI Z‐score and the 5‐year cumulative risk of relapse (HR: 1.07 [95% CI, 0.97%–1.17%], *p* = 0.18).

Table 2 shows the 5‐year EFS and OS among underweight, healthy‐weight, overweight, and obese patients according to clinical characteristics, risk group, MRD, and treatment protocol. The univariate analyses showed that within B‐lineage leukemia, the OS was 92.4% [95% CI, 86.7%–98.4%] in underweight patients, 89.0% [95% CI, 87.0%–91.1%] in healthy weight, and much lower at 83.2% [95% CI, 75.3%–92.0%] for overweight and 82.7% [95% CI, 76.8–89.0%] in obese patients (*p* = 0.03). Within patients with *BCR‐ABL1* fusion, the EFS was extremely poor in obese patients (0%), as compared to underweight (50.0% [95% CI, 12.5%–100%]), healthy‐weight (44.8% [95% CI, 31.4%–63.9%]), and overweight (50.0% [95% CI, 12.5%–100%]) patients (*p* < 0.001). The OS was also extremely poor in obese patients with *BCR‐ABL1* fusion (0%), as compared to underweight (50.0% [95% CI, 12.5%–100%]), healthy‐weight (66.1% [95% CI, 62.7%–83.0%]), and overweight (50.0% [95% CI, 12.5%–100%]) patients (*p* < 0.001). All six obese *BCR‐ABL1‐*positive patients were treated by SCMC‐ALL‐2005 protocol and none of them received tyrosine kinase inhibitor (TKI). Four of them relapsed, one had resistance disease and one died from toxicity. Among other clinical and biological subgroups, there were no significant differences in the 5‐year EFS or OS between healthy‐weight, underweight, overweight, and obese patients (Table 2).

Within the overall cohort, the independent factors associated with inferior EFS and OS were BCR‐ABL1 fusion (*p* < 0.001), MRD positivity by the end of induction (*p* < 0.001), and receiving SCMC‐ALL‐2005 protocol treatment (*p* < 0.001), and male sex (*p* < 0.001) (Table S1).

### 3.3 BMI and treatment‐related mortality and toxicities

Among the 1437 patients, 31 (2.2%) died of toxicities, including 22 infections (11 septic shock, 8 severe pneumonia, 1 central nervous system infection, 1 varicella infection, and 1 intestinal perforation), three hemorrhages, one intracranial hypertension, one renal failure, and four transplant‐related complications (2 graft failure, 1 hepatic veno‐occlusive disease, and 1 graft versus host disease).

**FIGURE 2:**
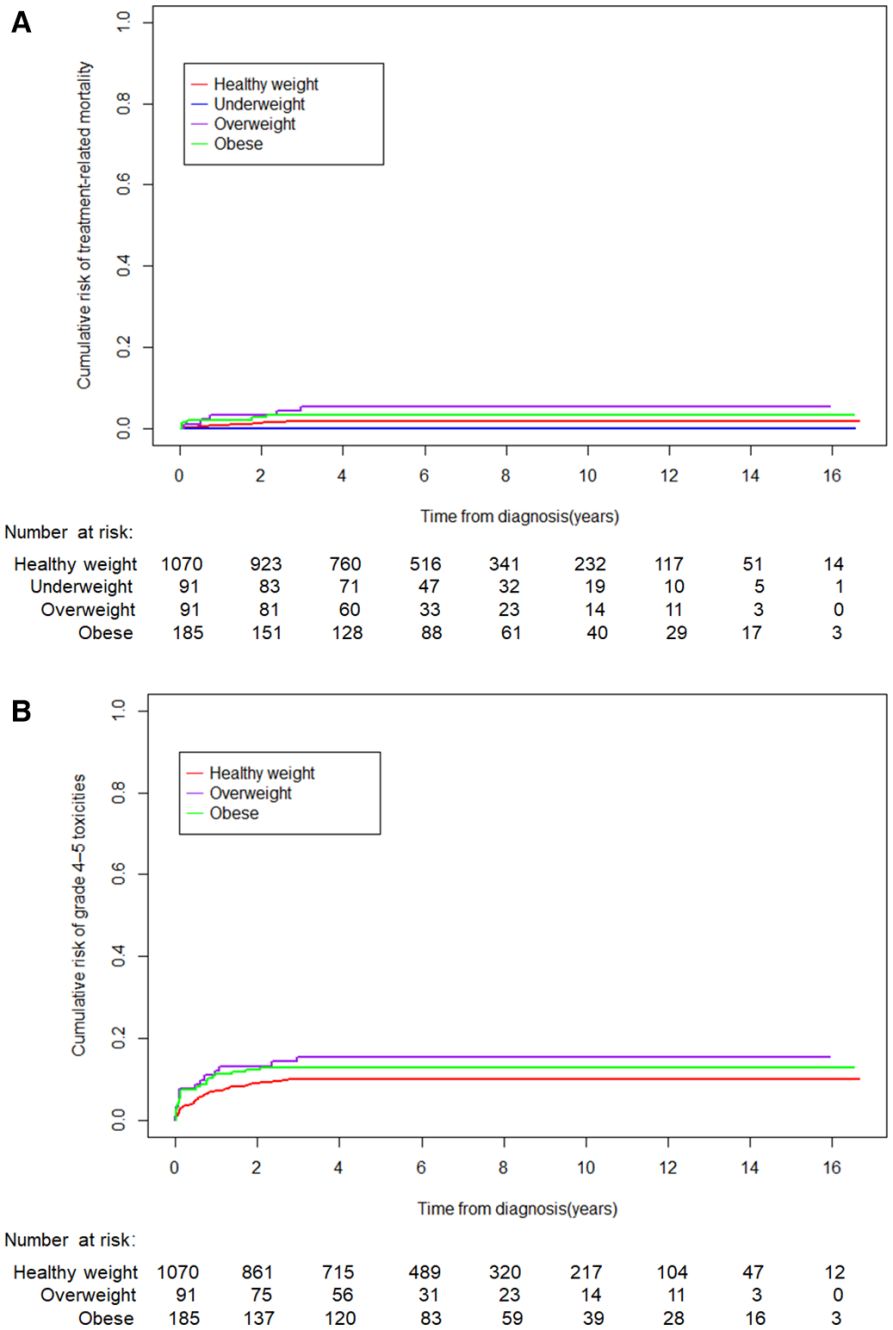
Cumulative incidence of the risk of treatment‐related mortality (A) and grade 4–5 toxicities (B).

Overweight and obesity were significantly associated with treatment‐related mortality (5.5% [95% CI, 0.8%–10.2%], 3.2% [95% CI, 0.6%–5.8%]) compared with underweight (0.0%) and healthy weight (1.9% [95% CI, 1.1%–2.7%]; *p* = 0.04; Figure 2A). In a multivariable analysis, being overweight (HR: 3.8 [95% CI, 1.3–11.4]; *p* = 0.02) and the SCMC‐ALL‐2005 protocol (HR: 5.6 [95% CI, 1.3–23.7]; *p* = 0.02) were associated with higher treatment‐related mortality (Table 3). As the SCMC‐ALL‐2005 was associated with higher mortality risk, a subgroup analysis was performed and showed that children who were overweight or obese had significantly higher treatment‐related mortality risk within the CCCG‐ALL‐2015 protocol but not the SCMC‐ALL‐2005 protocol (Table S2).

**TABLE 3:** Multivariable regression model of clinical characteristics and BMI groups associated with cumulative incidence of treatment‐related mortality

Variable	Cumulative incidence of treatment‐related mortality	
	Hazard ratio (95% CI)	*p* value
BMI groups		
Healthy weight	1	
Overweight	3.8 (1.3–11.4)	0.02
Obese	0.8 (0.2–3.6)	0.80
Final risk group		
Low		
Intermediate/High	1.4 (0.4–4.6)	0.59
Sex		
Female	1	
Male	1.1 (0.4–3.0)	0.83
Age at diagnosis		
<10 year	1	
≥10 years	2.2 (0.8–6.0)	0.12
Leukocyte count (×10^9^/L)		
<50	1	
≥50	1.5 (0.5–4.2)	0.44
Immunophenotype		
B‐lineage	1	
T‐lineage	1.0 (0.3–3.3)	0.98
MRD by the end of induction^a^		
<0.01%	1	
≥0.01%	1.6 (0.5–4.5)	0.42
Study protocol		
CCCG‐ALL‐2015	1	
SCMC‐ALL‐2005	5.6 (1.3–23.7)	0.02

Abbreviations: BMI, body mass index; MRD, minimal residual disease.

^a^ MRD test timing is on day 55 for patients treated with SCMC‐ALL‐2005 protocol, and on day 46 for patients treated with CCCG‐ALL‐2015 protocol.

The overall treatment‐related mortality (infection‐ and non‐infection‐related mortality) was higher in the overweight group than the healthy‐weight group (5.5% vs. 1.9%; *p* = 0.04). The infection‐related (3.3% vs. 1.4%; *p* = 0.16) and non‐infection‐related mortality rates (2.2% vs. 0.5%; *p* = 0.10) were marginally higher in the overweight group, as compared to the healthy‐weight group (Table S3).

Only healthy‐weight, overweight, and obese patients were included in the Common Terminology Criteria for Adverse Events grade 4–5 toxicity analysis (Table S4), as there were no events of treatment‐related mortality in underweight patients. The cumulative incidence of grade 4–5 toxicities trended lower in healthy‐weight patients (9.9% [95% CI, 8.1%–11.7%]) than in overweight (15.5% [95% CI, 8.0%–23.0%]) and obese patients (13.0% [95% CI, 8.1%–17.9%]; *p* = 0.13; Figure 2B). The rate of Grade 4–5 pancreatitis (0.3% vs. 1.1%) and Grade 4–5 hemorrhage (0.2% vs. 1.1%) were numerically higher in overweight/obese patients than in healthy‐weight patients, although the associations were not statistically significant (*p* = 0.10 and *p* = 0.06, respectively).

## 4 DISCUSSION

Obesity is a well‐established risk factor for mortality and relapse of certain cancers in adults. However, the evidence for the effects of obesity in pediatric leukemia remains inconsistent. This retrospective analysis evaluated the relationship between BMI at diagnosis and treatment outcomes in children with ALL. Our main analysis suggested that while weight status was not associated with survival, children who were overweight were at higher risk of treatment‐related mortality. There was also a significant trend between higher BMI Z‐score and poorer OS, as well as overall mortality. Taken together, our study concurs with the existing literature on the importance of weight control during diagnosis and treatment in children with leukemia.

To the best of our knowledge, this is one of the largest studies to examine the impact of weight status on OS, EFS, and mortality in the Asian population. Using BMI growth curves for Chinese children as a reference, the rate of obesity among Chinese children in this study (12.9%) is comparable to that of populations in the United States and Europe (13.0%–20.7%),^2^, ^4^, ^5^ but higher than that in a Japanese study (5.2%).^18^ Our future work includes validating our study findings with other Chinese and Asian cohorts of children with leukemia.

Overall, BMI groups at diagnosis did not significantly predict poorer OS, EFS, or relapse in our study. Inconsistent results have been reported in the literature. A recent meta‐analysis of 11 studies conducted in high‐income countries reported that pediatric ALL patients with a higher BMI had a 1.35 times higher risk of not achieving EFS than patients with a lower BMI; the association was present across all ages at diagnosis and leukemia phenotypes.^19^ On the other hand, our descriptive results suggested that patients who were underweight had better EFS and OS and no treatment‐related mortality. This interesting trend is contrary to other studies^5^, ^20^ that reported poorer outcomes in children with ALL who were underweight. Studies have reported that children tended to gain weight after induction and therapy due to metabolic effects of glucocorticoids.^21^ Hence, the small subset of patients who were underweight at diagnosis might be less vulnerable to the effects of weight gain during treatment. Unfortunately, we only had the BMI at diagnosis and could not draw any association between weight change and survival outcome. The specific nature of the association between BMI and treatment outcomes remains unclear.

At baseline, we did not identify differences in presenting features across the BMI groups. However, our stratified analysis suggested that the adverse effects of obesity on survival were more prominent in patients with certain risk factors, particularly B‐cell immunophenotype and *BCR/ABL1* mutation. This finding should be interpreted cautiously as the six obese patients with *BCR/ABL1* mutation were treated in the earlier era and did not receive TKIs, which is now recognized as an integral treatment for individuals with *BCR/ABL1* mutation. It may be possible that the effects of obesity may be more pronounced in patients who are already at risk for poorer survival outcomes due to the presence of specific genotype abnormalities and other risk factors.

After adjusting for clinically relevant factors, we found that patients who were overweight/obese at diagnosis had significantly higher treatment‐related mortality and trended toward lower OS than underweight and healthy‐weight patients. Overweight/obese patients may have immunological abnormalities that increase the likelihood of infection‐related death.^22^ Our study also contributes to growing evidence that overweight/obese patients are more likely to have a greater risk of grade 4–5 pancreatitis and hemorrhage than healthy‐weight patients, supporting the notion that BMI negatively impacts treatment‐related mortality. Denton et al.^23^ identified obesity as one of the predictors of pancreatitis in children and adolescents with ALL, which is consistent with our finding that overweight/obese patients had higher rates of pancreatitis. This may indicate that overweight/obese patients have less tolerance to severe adverse events. Both of our protocols did not specify calculation of chemotherapy doses according to ideal or actual, body weight. In our database, we identified nine obese patients with dosage adjustment based on their body surface area derived from the ideal weight. Subsequently, seven of them remained in remission, one relapsed and one died of treatment‐related toxicity. There was no consensus on chemotherapy dose adjustment for obese children.^24^ Prospective studies in this population are needed to standardize chemotherapy administration and to provide better supportive care in this special population.

Inconsistent findings on the effect of obesity in treatment outcomes may indicate that the pathophysiology of obesity's influence on leukemia is multifactorial and influenced by various factors.^19^, ^25^ Some older studies have proposed pharmacokinetic variation in the metabolism of chemotherapy drugs in obese patients.^26^, ^27^ However, support for this postulation has not been consistent in clinical studies.^3^ Others have proposed that nutritional status and health behaviors that influence weight might affect the patient's overall physiological response to cancer therapies and subsequent health outcomes.^28^ For example, one Korean study reported that the “spicy, fried meat, and fish” dietary pattern was associated with a higher risk of being overweight and all‐cause mortality than the “fish, egg, meat, fruits, and vegetables” dietary pattern.^29^ The seemingly discrepant results among these studies may be due to racial and ethnic differences in predisposing risk factors to obesity, responses to treatment, and lifestyle and behavioral factors.^19^, ^25^ We propose that future studies explore the interaction between genetic and non‐genetic (e.g., cultural and behavioral factors and diet) ethnic diversity in treatment responses to explain the variation in associations between weight and leukemia outcomes across studies in the literature.

Despite having a relatively large, well‐characterized sample with reliable sources of morbidity and mortality data, our findings should be considered in the context of several limitations. First, the patients in the study cohort were recruited from a single pediatric center in a relatively affluent urban city. Thus, it is unlikely to be a representative cohort, as China is a geographically large region with differences in resources between urban and rural areas. Hence, the rates of obesity and underweight, as well as the rate of mortality, are not generalizable to all Chinese children with ALL in the country. Second, this cohort consisted of patients who were diagnosed and treated for ALL from 2005 to 2018; there was a substantial improvement in the quality of medical and supportive care throughout the decade. Furthermore, we also found a significant association between overweight/obese and treatment‐related mortality within the CCCG‐ALL‐2015 protocol but not the earlier SCMC‐ALL‐2005 protocol; this finding suggested that the associations observed in the overall cohort might be driven by the ALL treatment regimens. Although we adjusted for demographic and clinical variables, other confounding factors not captured in this study might affect the association between weight and treatment outcomes. For example, socioeconomic status and behavioral factors, such as dietary intake and physical activity, were not assessed. Studies have also shown that weight management in children is a function of the family system^30^; therefore, future work should also evaluate the impact of family support and communal focus on weight control while the child is on active treatment.

## 5 CONCLUSIONS

While BMI status was not associated with EFS or OS in the overall cohort, we observed significant trends between higher BMI Z‐scores and poorer OS, and increased treatment‐related mortality. Chinese children with ALL who were overweight or obese also had a higher risk of treatment‐related mortality and developing severe toxicities. Weight control should be considered at diagnosis and early during treatment to reduce treatment‐related mortality in patients who are overweight/obese at diagnosis. In this regard, normalization of weight during the treatment period was found to mitigate the risk of lower EFS in a COG study.^2^

Our findings should be validated in larger multicenter studies to better understand the association between overweight/obesity at diagnosis and pediatric ALL outcomes.

## AUTHOR CONTRIBUTIONS

**Wenting Hu:** Formal analysis (lead); writing – original draft (equal). **Yin Ting Cheung:** Formal analysis (lead); methodology (equal); writing – original draft (equal). **Yanjing Tang:** Data curation (equal); investigation (equal); validation (equal). **Li Hong:** Investigation (equal); methodology (equal); supervision (equal). **Yuan Zhu:** Methodology (equal); resources (equal). **Jing Chen:** Investigation (equal); supervision (equal). **Zhuo Wang:** Investigation (equal); validation (equal). **Min Zhou:** Data curation (equal); validation (equal). **Yijin Gao:** Methodology (equal); validation (equal). **Jing Chen:** Methodology (equal); supervision (equal). **Benshang Li:** Investigation (equal); validation (equal). **Huiliang Xue:** Investigation (equal); supervision (equal). **Longjun Gu:** Investigation (equal); supervision (equal). **Shuhong Shen:** Investigation (equal); supervision (equal). **Jingyan Tang:** Investigation (equal); supervision (equal). **Ching‐Hon Pui:** Methodology (equal); supervision (equal); writing – review and editing (equal). **Hiroto Inaba:** Methodology (equal); writing – review and editing (equal). **Jiaoyang Cai:** Formal analysis (lead); methodology (equal); data curation (lead); writing – review and editing (equal).

## Supporting information


Table S1
Table S2
Table S3
Table S4
Figure S1

## Data Availability

Deidentified patient‐level data including presenting features, treatment response, and follow‐up, along with the study protocol are available by contacting Jiaoyang Cai (caijiaoyang@scmc.com.cn).
