# Supplementary material for: Association between body mass index at diagnosis and outcomes in Chinese children with newly diagnosed acute lymphoblastic leukemia
Source: Cancer Med. 2022 Sep 27;12(3):2850–60. doi: 10.1002/cam4.5188 (PMC9939171; doi:10.1002/cam4.5188)

Supplemental Table 1. Clinical and biological risk factors associated with event-free survival and overall survival (online-only)

| Variable | 5-year event-free survival | | 5-year overall survival | |
| --- | --- | --- | --- | --- |
|  | Hazard Ratio  (95% CI) | P Value | Hazard Ratio  (95% CI) | P Value |
| BMI groups |  |  |  |  |
| Healthy weight | 1 |  | 1 |  |
| Underweight | 0.9(0.5-1.4) | 0.57 | 0.9(0.5-1.8) | 0.77 |
| Overweight | 1.3(0.8-2.0) | 0.28 | 1.7(0.95-2.9) | 0.07 |
| Obese | 1.0(0.7-1.5) | 0.89 | 1.4(0.9-2.1) | 0.18 |
| Final Risk group |  |  |  |  |
| Low | 1 |  | 1 |  |
| Intermediate/ High | 1.3(0.97-1.8) | 0.08 | 1.5(0.99-2.4) | 0.06 |
| Sex |  |  |  |  |
| Female | 1 |  | 1 |  |
| Male | 1.6(1.2-2.1) | <0.001 | 1.3(0.9-1.8) | 0.14 |
| Age at Diagnosis |  |  |  |  |
| <10year | 1 |  | 1 |  |
| ≥10 years | 1.1(0.8-1.5) | 0.44 | 1.3(0.9-2.0) | 0.14 |
| Leukocyte count (x 10^9^/L) |  |  |  |  |
| <50 | 1 |  | 1 |  |
| ≥50 | 1.4(0.98-2.0) | 0.07 | 1.4(0.9-2.1) | 0.15 |
| Immunophenotype |  |  |  |  |
| B-lineage | 1 |  | 1 |  |
| T-lineage | 1.0(0.7-1.5) | 0.98 | 1.0(0.6-1.8) | 0.87 |
| t(9;22)(BCR-ABL1) |  |  |  |  |
| Absent | 1 |  | 1 |  |
| Present | 2.7(1.7-4.3) | <0.001 | 3.1(1.7-5.4) | <0.001 |
| t(12;21)(ETV6-RUNX1) |  |  |  |  |
| Present | 1 |  | 1 |  |
| Absent | 1.4(0.97-2.0) | 0.08 | 1.4(0.9-2.4) | 0.19 |
| MRD by the end of induction* |  |  |  |  |
| <0.01% | 1 |  | 1 |  |
| ≥0.01% | 2.4(1.8-3.1) | <0.001 | 2.0(1.4-2.8) | <0.001 |
| Study Protocol |  |  |  |  |
| CCCG-ALL-2015 | 1 |  | 1 |  |
| SCMC-ALL-2005 | 1.8(1.3-2.4) | <0.001 | 3.1(2.0-4.9) | <0.001 |

BMI, body mass index; MRD, minimal residual disease.

* MRD test timing is on day 55 for patients treated with SCMC-ALL-2005 protocol, and on day 46 for patients treated with CCCG-ALL-2015 protocol.

Supplemental Table 2. Cumulative risk of treatment‐related mortality according to BMI groups among patients treated with different protocols (online-only)

| Variable | 5-Year cumulative incidence of treatment‐related mortality  % (95% CI) | P Value |
| --- | --- | --- |
| BMI groups among SCMC-ALL-2005 protocol |  | 0.40 |
| Healthy weight | 2.8(1.6-4.0) |  |
| Overweight | 6.3(-0.7-13.3) |  |
| Obese | 3.1(0.1-6.1) |  |
| BMI groups among CCCG-ALL-2015 protocol |  | **0.003** |
| Healthy weight | 0.3(-0.2-0.8) |  |
| Overweight | 4.7(-1.7-11.1) |  |
| Obese | 3.7(-1.4-8.8) |  |

Supplemental Table 3. Treatment‐related mortality in healthy-weight and overweight/obese patients (online-only)

|  | Healthy weight  N(%) | Overweight  N(%) | P Value | Healthy weight  N(%) | Obese  N(%) | P Value |
| --- | --- | --- | --- | --- | --- | --- |
| Treatment‐related mortality (Infection) | 15(1.4) | 3(3.3) | 0.16 | 15(1.4) | 4(2.2) | 0.51 |
| Treatment‐related mortality (Non-infection) | 5(0.5) | 2(2.2) | 0.10 | 5(0.5) | 2(1.1) | 0.28 |
| Treatment‐related mortality ( Infection AND Non-infection) | 20(1.9) | 5(5.5) | **0.04** | 20(1.9) | 6(3.2) | 0.26 |
| No treatment-related mortality | 1050(98.1) | 86(94.5) | / | 1050(98.1) | 179(96.7) | / |

Supplemental Table 4. Comparison of grade 4/5 side effect between healthy-weight and overweight/obese patients

|  | Healthy weight  N(%) | Overweight/obese  N(%) | P Value |
| --- | --- | --- | --- |
| Total grade 4/5 side effect | 100(9.3) | 38 (13.8) | 0.03 |
| Grade 4/5 severe pneumonia | 42 (3.9) | 12 (4.3) | 0.73 |
| Grade 4/5 sepsis | 31 (2.9) | 10 (3.6) | 0.56 |
| Grade 4/5 tumor lysis syndrome | 12 (1.1) | 6 (2.2) | 0.24 |
| Grade 4/5 severe pancreatitis | 3 (0.3) | 3 (1.1) | 0.10 |
| Grade 4/5 severe hemorrhage | 2 (0.2) | 3 (1.1) | 0.06 |
| Grade 4/5 intestinal infection | 1 (0.1) | 2 (0.7) | 0.11 |
| Grade 4/5 seizure | 2 (0.2) | 1 (0.4) | 0.50 |
| Other grade 4/5 side effect | 7 (0.7) | 1 (0.4) | 0.99 |

Supplemental Figure 1. Growth curves of children and adolescents aged 0 to 18 in China.^14^ (online only)


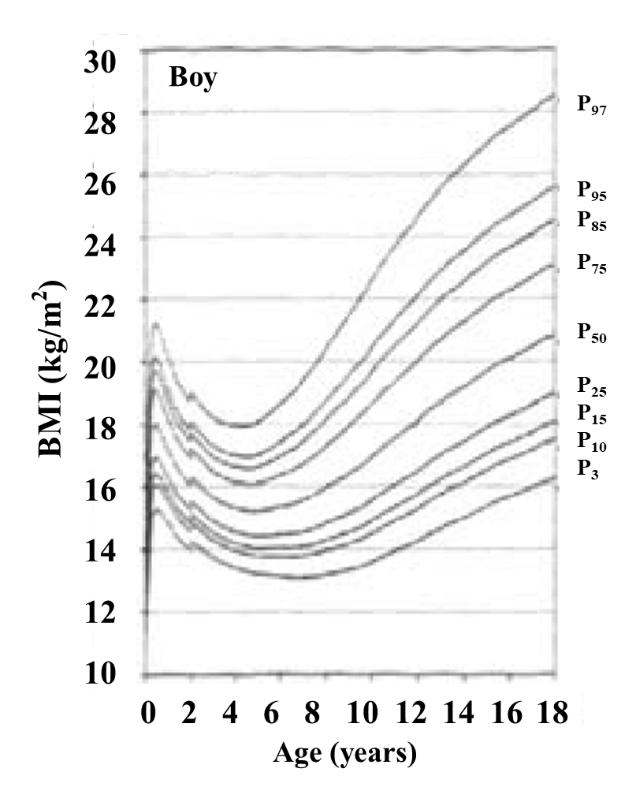

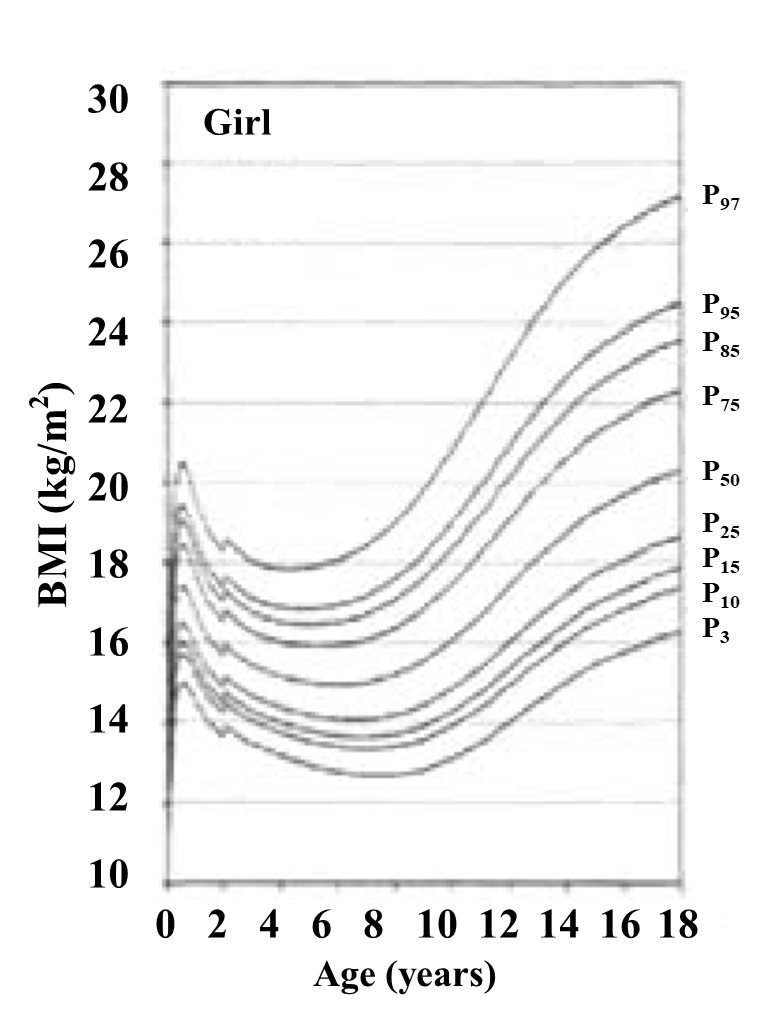

Supplement: Supplementary file 1 — Table S1 Table S2 Table S3 Table S4 Figure S1 [file CAM4-12-2850-s001.docx]
